# Outbreak of candidemia caused by fluconazole resistant *Candida parapsilosis* strains in an intensive care unit

**DOI:** 10.1186/s12879-016-1767-9

**Published:** 2016-08-20

**Authors:** Henrique Marconi Sampaio Pinhati, Luiz Augusto Casulari, Ana Carolina Remondi Souza, Ricardo Andreotti Siqueira, Camila Maria Gomes Damasceno, Arnaldo Lopes Colombo

**Affiliations:** 1 Ed. América Office Tower, SCN Q 1 BL F, - sala-1016, Asa Norte, Brasília, DF 70711-905 Brazil; 2 Universidade Federal de São Paulo, Rua Pedro de Toledo, 669, quinto andar, São Paulo, SP 04039-032 Brazil

**Keywords:** *Candida* sp, Candidemia, Fluconazole resistance, Antifungal treatment

## Abstract

**Background:**

Candidemia is an increasing problem in tertiary care hospitals worldwide. Here, we report the first outbreak of candidemia caused by fluconazole-resistant *C. parapsilosis* (FRCP) strains in Brazil.

**Methods:**

This was a cross-sectional study of clinical and microbiological data of all candidemic episodes diagnosed from July 2011 to February 2012 in a 200-bed tertiary care hospital. Initial yeast identification and susceptibility testing were performed using the VITEK 2 - System. Isolates of *Candida spp*. resistant to fluconazole were sent to a reference laboratory (LEMI-UNIFESP) for further molecular identification and confirmation of resistance by CLSI microdilution test. A multivariate analysis was conducted to identify factors associated with FRCP infection.

**Results:**

We identified a total of 40 critically ill patients with candidemia (15 women) with a median age of 70 years. The incidence of candidemia was 6 cases/1,000 patients admissions, including 28 cases (70 %) of infection with *C. parapsilosis*, 21 of which (75 %) were resistant to fluconazole. In only 19 % of FRCP candidemia cases had fluconazole been used previously. The results of our study indicated that diabetes is a risk factor for FRCP candidemia (*p* = 0.002). Overall, mortality from candidemia was 45 %, and mortality from episodes of FRCP infections was 42.9 %.

**Conclusions:**

The clustering of incident cases in the ICU and molecular typing of strains suggest horizontal transmission of FRCP. Accurate vigilant monitoring for new nosocomial strains of FRCP is required.

## Background

Hematogenous candidiasis is an alarming problem in tertiary care hospitals worldwide, especially in patients admitted in intensive care units (ICU) [1, 2]. Incidence rates of candidemia vary considerably among geographic areas and medical centers. For example, in Europe and the USA, different studies have reported incidence rates of 0.2–1.87 and 0–2.4 per 1,000 admissions, respectively [3, 4]. In Brazil and Latin America, nosocomial candidemia is an important problem in adults and children, and the reported incidence rates are 0.33–6 cases per 1,000 hospital admissions [5–7]. The large variation in the incidence rates of candidemia is probably due to a combination of several factors, including differences in resources available for medical care and training programs, difficulties in the implementation of infection control programs in hospitals in developing countries, limited number of health care workers to assist patients in critical care units, and less-aggressive practices of empirical antifungal therapy and prophylaxis for high-risk patients [6]. Hospital-acquired candidemia is associated with a large increase in hospitalization duration, attributable mortality (up to 40 %) and costs [8, 9].

Although *Candida albicans* is still considered the main causative species of candidemia worldwide, there is a concern over the rise in invasive infections caused by non-*albicans* species. In this regard, *Candida glabrata* has frequently been reported in Northern Europe and US medical centers [1, 10], whereas *Candida parapsilosis* and *Candida tropicalis* are the most common non-*albicans* species associated with fungemia in Latin America and Asia [11, 12]. Of note, a recent multicenter study in the USA found that *C. parapsilosis* was the second most commonly encountered non-*Candida albicans* species [13].

Fluconazole is accepted as an alternative treatment for patients with candidemia due to its efficacy and safety demonstrated by several clinical trials [14, 15]. For candidemia caused by *Candida parapsilosis* complex, in particular, fluconazole has been suggested as the best alternative for primary therapy [16]. There are some controversies in the literature regarding the effectiveness of echinocandins against infections of *C parapsilosis*, as high MICs are usually observed when this species is tested against drugs of this class [17].

**Fig. 1:**
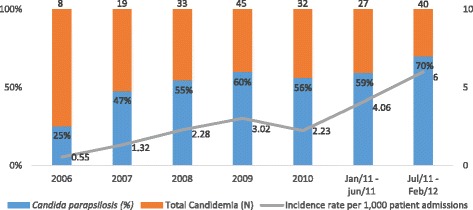
Incidence rates of candidemia and prevalence of *Candida parapsilosis* in ICU patients admitted at a single institution between 2006 and 2012

During July-2011 and February-2012, we observed a dramatic increase in the incidence of candidemia reported in ICU patients from a single institution compared to the previous period between January-2006 and June-2011, where 70 % of all episodes enrolled *C. parapsilosis* strains (Fig. 1). We were able to type 09 out 28 *C. parapsilosis* strains enrolled in this outbreak and they were considered genetically related [18]. Further antifungal susceptibility tests confirmed that most isolates were resistant to fluconazole. The present publication describes clinical and epidemiological data related to this outbreak of fluconazole resistant *C. parapsilosis* candidemia complementing information that was not explored in our previous report on molecular typing and resistance mechanisms related to the mentioned strains [18].

## Methods

We conducted a cross sectional study of clinical and laboratory data of all adult patients (age ≥18 years) diagnosed with candidemia during their hospitalization in intensive care unit between July, 2011 and February, 2012 (eight months period) to investigate a candidemia outbreak. Our local ethical committee approved the protocol (CEP: 73/2011).

### Laboratory procedures

Yeast identification and antifungal susceptibility testing were initially performed by the local microbiology laboratory using the VITEK 2 - system (bioMérieux, Marcy-I’Étoile, France). All *C. parapsilosis* isolates stored at the routine laboratory were sent to a reference laboratory (LEMI - Laboratório Especial de Micologia, Universidade Federal de São Paulo, São Paulo-SP, Brazil) for further molecular identification and confirmation of antifungal susceptibility testing by the Clinical and Laboratory Standards Institute (CLSI) reference method.

(i). Molecular identification of *C. parapsilosis* (*sensu lato*) isolates by real time TaqMan®PCR assays. DNA was extracted from the isolates using mechanical disruption with glass beads and phenol/chloroform [19]. Real-time PCR was performed using species-specific TaqMan® probes, as previously described by our group [20].
(ii). In vitro susceptibility testing. Antifungal susceptibility testing was performed at the reference laboratory using the CLSI microdilution assay [21]. Fluconazole and anidulafungin were provided by Pfizer Pharmaceutical Group (New York, NY, USA) and amphotericin B was provided by Sigma Chemical Corporation (St. Louis, MO, USA). The CLSI-M27-S4 document was used as the gold standard interpretative guidelines for classifying the *C. parapsilosis* (*sensu lato*) isolates as susceptible, susceptible dose dependent (SDD)/intermediate, or resistant to azoles and echinocandins [22].

### Statistical analysis

We performed univariate and multivariate analyses to identify risk factors associated with FRCP. For the univariate analysis, categorical variables were compared using the chi-square or Fisher exact tests, and continuous variables were compared using the Student t test or Mann–Whitney U test, as appropriate. All statistical tests were two-tailed, and the significance level was set at *p* < 0.05.

After the initial univariate analysis, the variables with significant FRCP association were included in a multivariable analysis with adjustments for the covariates of age and gender. A Poisson regression with robust variance (linear-log) was employed for the multivariate analysis. The level of significance was set at *p* < 0.05 [23].

## Results

During the study period, 40 episodes of candidemia were reported in ICU patients. The median age of the patients was 70 years (range: 23–91 years), and 37.5 % of the patients were female. The overall incidence of candidemia during the eight month study period was 6 cases/1,000 patients-day admissions.

The VITEK 2 system identified 28 blood stream *Candida* isolates as *C. parapsilosis* (70 %), 9 as *C. albicans* (22.5 %), 2 as *C. glabrata* (5 %) and 1 as *C. tropicalis* (2.5 %). Using the VITEK 2 system, fluconazole resistance or SDD was detected in 21 out 28 *C. parapsilosis* (75 %) isolates tested. All 9 *C. albicans*, 2 *C. glabrata* and 1 *C. tropicalis strains* were considered susceptible to fluconazole by the automated system. All 40 *Candida* spp strains were susceptible to amphotericin B and anidulafungin.

Fourteen out 28 clinical strains of *C. parapsilosis* isolates enrolled into the outbreak had been stored by the routine laboratory and were available to be sent to the reference laboratory (LEMI-UNIFESP) for further molecular identification and confirmation of antifungal susceptibility test results by the CLSI broth microdilution (BMD) reference method. Except for one clinical isolate identified as *C. metapsilosis*, all other strains were genetically identified as *C. parapsilosis* (*sensu stricto*) when tested using species specific TaqMan® probes. Despite some differences among MICs generated by the two methods, the categorical agreement between VITEK 2 and CLSI BMD was very good (72 %). A total of 11 out 14 *C parapsilosis* strains categorized as SDD or resistant to fluconazole by the VITEK 2 system, were considered resistant or SDD when tested by the CLSI method. Of note, 9 of the 11 strains categorized as resistant to fluconazole by the BMD exhibited similar genetic profile by microsatellite assay and molecular mechanisms related to azole resistance as reported by our group elsewhere [18]. No strains of *C. albicans*, *C. tropicalis* or *C. glabrata* were available for testing by the CLSI methodology. No strains of *C. parapsilosis* were resistant to anidulafungin or amphotericin B as already documented by the VITEK 2 system.

Considering that only 14 of the 40 isolated strains were tested by the CLSI method, we used the information provided by the VITEK 2 system to check for risk factors associated with infection by fluconazole-SDD or -resistant *Candida*.

The epidemiological and clinical characteristics of the patients infected by FRCP strains and those infected by fluconazole-susceptible *Candida* (FSC) species are presented in Table 1. In general, most demographic and clinical data were similar between the two patient groups. The exposition to central venous catheter, hemodialysis, surgery, mechanical ventilation, antibiotics and corticosteroids, were similar in both groups. It is important to mention that only 4 of the 21 patients (19 %) infected by FRCP had been previously exposed to fluconazole before developing candidemia compared to 3 of the 19 patients (15.8 %) infected by FSC strains (*p* = 1). After univariate analysis, only 2 variables were identified as independent factors associated with infection by FRCP: diabetes mellitus (*p* = 0.013) and parenteral nutrition (*p* = 0.059). After multivariate analysis, only diabetes remained an independent factor associated with infection by FRCP (*p* = 0.001 OR 1.3–3.5).

**Table 1:** Epidemiological and clinical characteristics of 40 ICU patients with fungemia caused by fluconazole-resistant *C. parapsilosis* (FRCP) and fluconazole-susceptible *Candida* species (FSC)

	FRCP (*N* = 21)	FSC^a^ (*N* = 19)	*P value*
Age (years) - median (range)	70 (23–91)	76 (35–90)	0.311
Gender female - n (%)	7 (33.3)	8 (42.1)	0.567
Time to candidemia – median/range	22 (0–83)	25 (7–134)	0.185
Apache II score – median/range	14 (3–29)	16 (3–30)	0.138
Death up to 30 days after candidemia (%)	9 (42.9)	9 (47.4)	0.78
Comorbidities - n (%)			
Cancer	5 (23.8)	2 (10.5)	0.270
Pulmonary disease	9 (42.9)	11 (57.9)	0.342
Cardiac disease	6 (28.6)	3 (15.8)	0.557
Diabetes mellitus^b^	11 (52.4)	2 (10.5)	0.013
Renal failure	4 (19.0)	3 (15.8)	1.000
Previous exposure to medical procedures and medicines - n (%)			
Short-term catheter	21 (100.0)	17 (89.5)	0.424
Catheter tip positive	4 (19.0)	3 (15.8)	1.000
Previous hemodialysis	3 (14.3)	1 (5.3)	0.673
Prior surgery	12 (61.9)	10 (52.6)	0.775
Mechanical ventilation	15 (71.4)	14 (73.7)	0.873
Parenteral nutrition	7 (33.3)	1 (5.3)	0.059
Previous Hospitalization	4 (19)	8 (42.1)	0.112
Antibiotics	10 (47.6)	8 (42.1)	0.726
Steroids	8 (38.1)	7 (36.8)	0.942
Any antifungal drug	7 (33.3)	3 (15.8)	0.361
Fluconazole	4 (19.0)	3 (15.8)	1.000

^a^all *Candida* strains considered susceptible to fluconazole by the Vitek 2 system. ^b^variable identified as independent factor associated with infection by FRCP

The overall candidemia mortality rates at 30 days after diagnosis were 45 % (18/40) for all patients, 47.4 % (9/19) for the group infected by FSC and 42.9 % (9/21) for the group infected by FRCP (*p* = 0.8). Of note, liposomal amphotericin B was the major therapeutic option employed for the treatment of patients infected by FRCP (12 out 21 patients, 57 %).

## Discussion

This study is the first to describe an outbreak of candidemia caused by fluconazole-resistant *C. parapsilosis* strains in ICU patients of a tertiary care hospital in Brazil. It is important to note that the *C. parapsilosis* strains were identified by molecular methods and the phenotype of resistance to fluconazole was examined by 2 different methods, including the CLSI BMD assay.

The outbreak of *C. parapsilosis* was confirmed by a sharp increase in the incidence rate of candidemia reported in the ICU as well as by molecular typing of the isolates by microsatellite technique (please, see data about molecular typing on reference Souza et al. 2015).

During the study period, hospital physicians worked under the reasonable assumption that most episodes of *C. parapsilosis* candidemia were completely refractory to treatment with fluconazole (personal communication). As a consequence, the hospital staff decided to use liposomal amphotericin B (L-AMB) as the antifungal drug of choice to treat all episodes of *C. parapsilosis* candidemia. This decision was based on the putative limitations of echinocandins against *C. parapsilosis* infections and the good activity of L-AMB against biofilm-producing strains of *Candida* species [14, 24].

Despite treatment with L-AMB, the crude mortality rate of candidemia due to fluconazole resistant *C. parapsilosis* was 42.9 %. Indeed, this finding contradicts the current consensus that the mortality rate due to infection with *C. parapsilosis* is usually lower than those due to infection with *C. albicans* or *C. tropicalis* [13]. The high mortality rates observed with our casuistic study of FRCP candidemia is probably secondary to the high percentage of elderly patients (70 % >60 years) as well as the high Apache II score related to those patients (see Table 1).

Although uncommon, invasive infections due to FRCP isolates have been increasingly reported. In accordance with recent studies, using antifungal susceptibility tests that are considered gold standards (CLSI and EUCAST- European committee on Antimicrobial Susceptibility Testing, microbroth assays), the rates of fluconazole resistance in blood stream *C. parapsilosis* isolates ranges from 3.4 to 7.5 % in the USA [17, 25, 26], 0 to 6.3 % in Europe and up to 5.4 in Asia [17, 27, 28].

In Latin America, *C. parapsilosis* blood stream isolates have rarely been reported to be resistant to fluconazole. A recent study evaluating a total of 672 episodes of candidemia in 21 medical centers from 7 countries in Latin America found that the fluconazole MIC value for 90 % of 178 strains of *C parapsilosis* was 1.0 mg/mL, and only 2 strains exhibited susceptibility-dose dependence against this drug [5]. In Brazil, the largest multicenter study ever published on the epidemiology of candidemia evaluated a total of 712 episodes of candidemia and found that all 146 *C. parapsilosis* strains tested were susceptible to azoles [7]. Based on those studies we can state with certainty that the present series of FRCP candidemia represents the largest experience with this emergent pathogen in our region.

*C. parapsilosis* (*sensu stricto*) has been described as the most prevalent *C. parapsilosis* complex species in both superficial and invasive human infections, and *C. orthopsilosis* and *C. metapsilosis* are responsible for less than 10 % of the *C. parapsilosis* group infections in reported in different series [29–35]. Accordingly, 13 out of 14 of our *C. parapsilosis* isolates available for molecular identification were identified as *C. parapsilosis* (*sensu stricto*).

It is well established that *C. parapsilosis* is a commensal of human skin that can be transmitted horizontally via contaminated external sources and hands of health care workers [36]. We tried to identify risk factors associated with episodes of candidemia caused by FRCP, but our findings were limited by the small population size and casuistic nature of this study as well as the absence of environmental sampling data and the absence of a survey of *Candida* colonization among patients and health care workers. By multivariate analysis, diabetes was the only medical condition found to be independently associated with FRCP. In the present study, central catheters were not associated with FRCP; furthermore, they were almost universally present in the patients who developed candidemia, regardless of whether the causative *Candida* strain was susceptibility to fluconazole (97.5 %, 39 of 40 episodes). However, considering that most patients with FRCP fungemia were not previously exposed to fluconazole, we still propose that the present outbreak was probably a series of catheter-related fungemias that occurred when a resistant strain of *C parapsilosis* was exogenously acquired by the patients during their hospitalization (15 to 40 days before developing candidemia) through contact with health care workers that were putatively colonized by this emergent pathogen.

The identification of diabetes as a risk factor of infection by FRCP in our series may be explained by two aspects: (i) this medical condition may increase the number of opportunities and the intensity of physical contact between health care workers and patients to appropriately monitor this metabolic disease both clinically and through laboratory tests; (ii) diabetic patients are more prone to colonization by *S. aureus* and eventually *Candida*, facilitating a putative episode of colonization by a hospital acquired fluconazole-resistant *Candida* strain.

## Conclusion

We were able to report the first outbreak of fluconazole resistant *C parapsilosis* strains involving adult ICU patients without a previous history of long exposition to triazoles. The clustering of incident cases in the ICU and molecular typing of strains strongly suggested the horizontal transmission of FRCP. Despite apparently uncommon, resistance of *C parapsilosis* to fluconazole has been increasingly reported and accurate vigilant monitoring for new nosocomial strains of FRCP is required.

## Abbreviations

BMD, broth microdilution; CLSI, Clinical and Laboratory Standards Institute; EUCAST, European committee on Antimicrobial Susceptibility Testing; FRCP, fluconazole-resistant *C. parapsilosis*; FSC, fluconazole-susceptible *Candida*; ICU, intensive care unit; L-AMB, Liposomal amphotericin B

## References

[1] Falagas ME, Roussos N, Vardakas KZ (2010). Relative frequency of *albicans* and the various non-*albicans Candida* spp among candidemia isolates from inpatients in various parts of the world: a systematic review. Int J Infect Dis.

[2] Pfaller MA, Messer SA, Moet GJ, Jones RN, Castanheira M (2011). *Candida* bloodstream infections: comparison of species distribution and resistance to echinocandin and azole antifungal agents in Intensive Care Unit (ICU) and non-ICU settings in the SENTRY Antimicrobial Surveillance Program (2008–2009). Int J Antimicrob Agents.

[3] Yapar N (2014). Epidemiology and risk factors for invasive candidiasis. Ther Clin Risk Manag.

[4] Pfaller MA, Diekema DJ (2007). Epidemiology of invasive candidiasis: a persistent public health problem. Clin Microbiol Rev.

[5] Nucci M, Queiroz-Telles F, Alvarado-Matute T, Tiraboschi IN, Cortes J, Zurita J (2013). Epidemiology of candidemia in Latin America: a laboratory-based survey. PLoS One.

[6] Moretti ML, Trabasso P, Lyra L, Fagnani R, Resende MR, de Oliveira Cardoso LG (2013). Is the incidence of candidemia caused by *Candida glabrata* increasing in Brazil? Five-year surveillance of *Candida* bloodstream infection in a university reference hospital in southeast Brazil. Med Mycol.

[7] Colombo AL, Nucci M, Park BJ, Nouér SA, Arthington-Skaggs B, da Matta DA (2006). Epidemiology of Candidemia in Brazil: a nationwide sentinel surveillance of candidemia in eleven medical centers. J Clin Microbiol.

[8] Heimann SM, Cornely OA, Wisplinghoff H, Kochanek M, Stippel D, Padosch SA (2014). Candidemia in the intensive care unit: analysis of direct treatment costs and clinical outcome in patients treated with echinocandins or fluconazole. Eur J Clin Microbiol Infect Dis.

[9] Falagas ME, Apostolou KE, Pappas VD (2006). Attributable mortality of candidemia: a systematic review of matched cohort and case-control studies. Eur J Clin Microbiol Infect Dis.

[10] Pfaller MA, Diekema DJ, Gibbs DL, Newell VA, Ellis D, Tullio V (2010). Results from the Artemis DISK Global Antifungal Surveillance Study, 1997 to 2007: a 10.5-year analysis of susceptibilities of *Candida* species to Fluconazole and Voriconazole as determined by CLSI standardized disk diffusion. J Clin Microbiol.

[11] Hinrichsen SL, Falcão É, Vilella TAS, Colombo AL, Nucci M, Moura L (2008). Candidemia in a tertiary hospital in northeastern Brazil. Rev Soc Bras Med Trop Agosto De.

[12] Morii D, Seki M, Binongo JN, Ban R, Kobayashi A, Sata M, Hashimoto S, Shimizu J, Morita S, Tomono K (2014). Distribution of *Candida* species isolated from blood cultures in hospitals in Osaka. Japan J Infect Chemother.

[13] Pfaller MA, Andes DR, Diekema DJ, Horn DL, Reboli AC, Rotstein C (2014). Epidemiology and outcomes of invasive candidiasis due to non-*albicans* species of *Candida* in 2,496 patients: data from the Prospective Antifungal Therapy (PATH) Registry 2004–2008. PLoS One.

[14] Cornely OA, Bassetti M, Calandra T, Garbino J, Kullberg BJ, Lortholary O (2012). ESCMID* guideline for the diagnosis and management of *Candida* diseases 2012: non-neutropenic adult patients. ClinMicrobiol Infect.

[15] Colombo AL, Guimarães T, Camargo LF, Richtmann R, de Queiroz-Telles F, Salles MJ (2013). Brazilian guidelines for the management of candidiasis - a joint meeting report of three medical societies: Sociedade Brasileira de Infectologia, Sociedade Paulista de Infectologia and Sociedade Brasileira de Medicina Tropical. Braz J Infect Dis.

[16] Pappas PG, Kauffman CA, Andes D, Benjamin DK, Calandra TF, Edwards JE (2009). Clinical practice guidelines for the management of candidiasis: 2009 update by the Infectious Diseases Society of America. Clin Infect Dis.

[17] Pfaller MA, Messer SA, Woosley LN, Jones RN, Castanheira M (2013). Echinocandin and triazole antifungal susceptibility profiles for clinical opportunistic yeast and Mold isolates collected from 2010 to 2011: application of New CLSI clinical breakpoints and epidemiological Cutoff values for characterization of geographic and temporal trends of antifungal resistance. J ClinMicrobiol.

[18] Souza ACR, Fuchs BB, Pinhati HMS, Siqueira RA, Hagen F, Meis JF (2015). *Candida parapsilosis* Resistance to Fluconazole: Molecular Mechanisms and In Vivo Impact in Infected Galleria mellonella Larvae. Antimicrob Agents Chemother.

[19] Jain P, Khan ZK, Bhattacharya E, Ranade SA (2001). Variation in random amplified polymorphic DNA (RAPD) profiles specific to fluconazole-resistant and -sensitive strains of *Candida albicans*. Diagn Microbiol Infect Dis.

[20] Souza ACR, Ferreira RC, Gonçalves SS, Quindós G, Eraso E, Bizerra FC (2012). Accurate identification of *Candida parapsilosis* (sensu lato) by use of mitochondrial DNA and real-Time PCR. J Clin Microbiol.

[21] Clinical and Laboratory Standards Institute (2008). Reference method for broth dilution antifungal susceptibility testing of yeasts; approved standard-3rd ed. document M27-A3.

[22] Clinical and Laboratory Standards Institute (2012). Reference method for broth dilution antifungal susceptibility testing of yeasts. 4th informational supplement.document M27-S4.

[23] Barros AJ, Hirakata VN (2003). Alternatives for logistic regression in cross-sectional studies: an empirical comparison of models that directly estimate the prevalence ratio. BMC Med Res Methodol.

[24] Tumbarello M, Posteraro B, Trecarichi EM, Fiori B, Rossi M, Porta R (2007). Biofilm production by *Candida* species and inadequate antifungal therapy as predictors of mortality for patients with candidemia. J Clin Microbiol.

[25] Cleveland AA, Farley MM, Harrison LH, Stein B, Hollick R, Lockhart SR (2012). Changes in incidence and antifungal drug resistance in candidemia: results from population-based laboratory surveillance in Atlanta and Baltimore, 2008–2011. Clin Infect Dis.

[26] Pfaller MA, Jones RN, Castanheira M (2014). Regional data analysis of *Candida non*-*albicans* strains collected in United States medical sites over a 6-year period, 2006–2011. Mycoses.

[27] Arendrup MC, Dzajic E, Jensen RH, Johansen HK, Kjældgaard P, Knudsen JD (2013). Epidemiological changes with potential implication for antifungal prescription recommendations for fungaemia: data from a Nationwide fungaemia surveillance programme. Clin Microbiol Infect.

[28] Minea B, Nastasa V, Moraru RF, Kolecka A, Flonta MM, Marincu I (2015). Species distribution and susceptibility profile to fluconazole, voriconazole and MXP-4509 of 551 clinical yeast isolates from a Romanian multi-centre study. Eur J Clin Microbiol Infect Dis.

[29] Silva AP, Miranda IM, Lisboa C, Pina-Vaz C, Rodrigues AG (2009). Prevalence, distribution, and antifungal susceptibility profiles of *Candida parapsilosis*, *C. orthopsilosis*, and *C. Metapsilosis* in a tertiary care hospital. J Clin Microbiol.

[30] Gonçalves SS, Amorim CS, Nucci M, Padovan ACB, Briones MRS, Melo ASA (2010). Prevalence rates and antifungal susceptibility profiles of the *Candida parapsilosis* species complex: results from a nationwide surveillance of candidaemia in Brazil. Clin Microbiol Infect.

[31] Ruiz LS, Khouri S, Hahn RC, da Silva EG, de Oliveira VK, Gandra RF (2013). Candidemia by species of the *Candida parapsilosis* complex in children’s hospital: prevalence, biofilm production and antifungal susceptibility. Mycopathologia.

[32] Bonfietti LX, Martins Mdos A, Szeszs MW, Pukiskas SB, Purisco SU, Pimentel FC (2012). Prevalence, distribution and antifungal susceptibility profiles of *Candida parapsilosis*, *Candida orthopsilosis* and *Candida metapsilosis* bloodstream isolates. J Med Microbiol.

[33] Trabasso P, Matsuzawa T, Fagnani R, Muraosa Y, Tominaga K, Resende MR (2015). Isolation and drug susceptibility of *Candida parapsilosis* sensu lato and other species of *C. parapsilosis* complex from patients with blood stream infections and proposal of a novel LAMP identification method for the species. Mycopathologia.

[34] Cantón E, Pemán J, Quindós G, Eraso E, Miranda-Zapico I, Álvarez M (2011). Prospective multicenter study of the epidemiology, molecular identification, and antifungal susceptibility of *Candida parapsilosis*, *Candida orthopsilosis*, and *Candida metapsilosis* isolated from patients with candidemia. Antimicrob Agents Chemother.

[35] Feng X, Ling B, Yang G, Yu X, Ren D, Yao Z (2012). Prevalence and distribution profiles of *Candida parapsilosis*, *Candida orthopsilosis* and *Candida metapsilosis* responsible for superficial candidiasis in a Chinese university hospital. Mycopathologia.

[36] Trofa D, Gácser A, Nosanchuk JD (2008). *Candida parapsilosis*, an emerging fungal pathogen. Clin Microbiol Rev.

